# Quality of Life in Survivors of a Primary Bone Tumour: A Systematic Review

**DOI:** 10.1080/13577149977622

**Published:** 1999-12

**Authors:** Christine Eiser, Robert J. Grimer

**Affiliations:** 1 CRC Child and Family Research Group School of Psychology University of Exeter Exeter EX4 4QG UK; 2 Royal Orthopaedic Hospital Oncology Service Royal Orthopaedic Hospital NHS Trust Woodlands, Northfield Birmingham B 31 4111 UK; 3 CRC Child and Family Research Group Department of Psychology University of Sheffield Western Bank Sheffield S10 2TP UK

## Abstract

*Purpose.* We conducted a systematic search of published literature, to assess (i) quality of life (QoL) for survivors of a bone tumour compared with the normal population; (ii) QoL implications following amputation, successful or failed limb salvage; (iii) adaptation of young children to amputation compared with older children or adolescents.

*Methods.* Electronic databases were searched including Medline, PsycLIT and Cinahl covering the years 1982– 1998.

*Results.* We identified 11 studies. Regardless of treatment, physical functioning was poor compared with population norms or healthy siblings.There was less consistent evidence regarding emotional functioning. Seven studies compared functioning in amputees and limb salvage patients.Two reported advantages in physical function for the limb salvage group, one for the amputees and the rest no differences. Evidence about social functioning or marriage is inconclusive, but there are suggestions that amputees report more job discrimination.

*Discussion.* The literature is inconclusive, largely because of methodological problems. These include small and non-representative samples, and lack of sensitive and appropriate measures. Specific gaps in the literature include very little work concerned with psychological outcomes for children, or for those experiencing failed limb salvage. More attention needs to be given to gender differences in emotional response to traumatic surgery.The implications of the results for helping families balance the merits of different treatments are discussed.


Sarcoma (1999) 4, 183–190

ORIGINAL ARTICLE

### Quality of life in survivors of a primary bone tumour: a systematic review

CHRISTINE EISER[1] & ROBERT J. GRIMER[2]

[1]*CRC Child and Family Research Group, School of Psychology, University of Exeter, Exeter EX4 4QG,* [2]*Royal Orthopaedic Hospital Oncology Service, Royal Orthopaedic Hospital NHS Trust, Woodlands, Northfield, Birmingham B31 4111, UK*

**Abstract**

*Purpose.* We conducted a systematic search of published literature, to assess (i) quality of life (QoL) for survivors of a bone tumour compared with the normal population; (ii) QoL implications following amputation, successful or failed limb salvage; (iii) adaptation of young children to amputation compared with older children or adolescents.

*Methods.* Electronic databases were searched including Medline, PsycLIT and Cinahl covering the years 1982–1998.

*Results.* We identified 11 studies. Regardless of treatment, physical functioning was poor compared with population norms or healthy siblings. There was less consistent evidence regarding emotional functioning. Seven studies compared functioning in amputees and limb salvage patients. Two reported advantages in physical function for the limb salvage group, one for the amputees and the rest no differences. Evidence about social functioning or marriage is inconclusive, but there are suggestions that amputees report more job discrimination.

*Discussion.* The literature is inconclusive, largely because of methodological problems. These include small and non-representative samples, and lack of sensitive and appropriate measures. Specific gaps in the literature include very little work concerned with psychological outcomes for children, or for those experiencing failed limb salvage. More attention needs to be given to gender differences in emotional response to traumatic surgery. The implications of the results for helping families balance the merits of different treatments are discussed.

### Introduction

The issue of quality of life (QoL) following treatment for cancer has been extensively discussed. QoL may be compromised by physical complications associated with the initial disease or late side-effects of chemotherapy. QoL may also be compromised psychologically as a result of concerns about relapse or recurrence. Survivors may experience prejudice and limited work opportunities, restricted social lives or opportunities to travel. For these reasons, there have been a number of follow-ups of survivors of cancer generally, and there is now considerable evidence relating physical and psychological sequaelae to radiotherapy or specific chemotherapy protocols.[1]

In addition to the difficulties experienced by survivors of any cancer, those treated for a bone tumour may experience additional threats to QoL as a consequence of restricted mobility, pain and stigmatization. With modern chemotherapy, survival rates approaching 60–65% can now be achieved for patients with bone tumours. Some 85% are offered limb salvage surgery and the remainder amputation. Patients treated with limb salvage have been shown to have a slightly higher rate of recurrence compared with those treated by amputation, although overall survival rates are comparable.[2] However, endoprostheses can fail with the result that many patients may ultimately need amputation.

The decision to amputate or treat by limb salvage is complex. Neither treatment appears to be associated with improved survival. Amputation may be the preferred option for those who wish to participate in many physical activities including contact sports (an artificial limb can relatively easily be replaced). In contrast, limb salvage has the merit of preserving body image, and the decision to amputate can subsequently be taken at any time. However, salvaged limbs vary in function, and many patients are so concerned about breakages that they adopt very sedentary life-styles. Wherever it is practically possible to perform limb salvage, this option is likely to be preferred by clinicians, parents and patients, as well as being less expensive than amputation.[41] Failed limb salvage procedures are potentially problematic for patients emotionally, in that they may feel that they have failed their surgeon in some way, that they have contributed personally to the failure through their own recklessness or that the years of struggling

Correspondence to: Christine Eiser, PhD, CRC Child and Family Research Group, Department of Psychology, University of Sheffield, Western Bank, Sheffield S10 2TP, UK. Tel: (0114) 2226536; Fax: (0114) 2766515; Email: C.Eiser@sheffield.ac.uk

1357-714X print/1369-1643 online/99/040183-08 © 1999 Taylor & Francis Ltd with the endoprosthesis amounted to a waste of time.

The concept of QoL is itself elusive, dismissed by some as 'a kind of umbrella under which are placed many different indexes with whatever the user wants to focus on'.[3] For others, QoL is a necessary and important outcome measure offering a balance to earlier emphases on survival statistics and morbidity.[4,5] There is a general consensus that QoL is a multidimensional concept involving physical, psychological and social elements and most measures include domains to assess each of these separately. Critically, QoL assessments must include information from the patient about their own perceptions of the impact of disease on their lives.

The demand for measures of QoL has far outweighed the level of sophistication of research or theory in the area: 'The term "health-related quality of life" was coined as a way of justifying the use of currently available measures under a new banner'.[6] The emphasis is very much on functional capacity, reflecting the traditional approach to assessing medical outcomes. Thus the focus is on the ability to perform everyday tasks and fulfil occupational and social roles. This focus on functional capacity ignores the relative meaning of these tasks to the individual, and assumes there is an optimal level of functioning to which everyone aspires. In order to balance this functional bias, QoL measures need to include some assessment of the meaning of the illness to the individual.

Since there are currently few comprehensive measures of health-related QoL available, it is necessary to rely on proxy measures, including for example physical function (physical symptoms and pain); psychological function (anxiety, depression or body image); and social function (employment, social relations or marital status).

While there may be disagreements about measurement, the issue of QoL is of central concern both to surgeons, patients and their families. Clinically, a number of questions may be raised on a daily basis.

- How does treatment for a bone tumour affect an individual's QoL?
- How does QoL differ between amputees, successful limb salvage and failed limb salvage?
- Do young children adapt better to amputation than older children or adolescents?

This paper reports the results of a systematic search of current literature with the aim of assessing current findings relating to these questions. In addition, methodological problems are identified and recommendations made about future work.

### Method

The following electronic databases were searched: Medline, PsycLIT and Cinahl covering the years 1982–1998. Search terms included QoL, health status, functional status, well-being, bone tumour, osteosarcoma, Ewing's tumour and were subsequently modified to meet the requirements of the different databases. We also examined reference lists in reviews to identify other relevant studies. A data extraction sheet was used to summarize the methods and results and the data were organized using Reference Manager.

### *Inclusion and exclusion criteria*

Given the limited research in the area, no restrictions were placed on study design or methods. However, papers which reported clinical or survival data but no psychological data were excluded, as were descriptive accounts which did not include any statistical analyses. Papers were included which involved statistical comparisons (1) with groups (e.g. healthy siblings) or population norms, and (2) between groups undergoing different treatment options (amputation or limb salvage).

### Results

We identified 11 studies, which are summarized chronologically in Table 1.[18–39] Methods included comparisons with population norms, well siblings, and between amputee and limb salvage patients. Eight of the 11 studies made comparisons against population norms. Both Nicholson *et al.*[9] and Novakovic *et al.*[15] included a comparison group of well siblings. One study only involved comparisons between amputees and limb salvage groups.[17]

### How does treatment for a bone tumour affect an individual's QoL?

### *Physical functioning*

Seven studies attempted to measure physical function. Three used the Functional Evaluation of Reconstructive Procedures,[32] and measures by Butterfield (unpublished) or Karnofsky[36] were each used in one study. The physical function subscale of the SF36[35] was used in one study.[14] Three studies assessed function on the basis of a semi-structured interview.[10,11,14]

In the two studies where comparisons were made with healthy siblings,[9,15] there was evidence that the bone tumour group had poorer physical function than their siblings. These included specific difficulties climbing stairs,[9] and 'general physical function'.[15] Thirty patients treated by limb salvage showed significant problems on a number of domains including physical functioning and physical role performance, pain, general health and social functioning compared with British norms.[14]

### *Emotional functioning*

Four studies reported no differences between survivors and population norms[8,11,12,14] based on standardized measures of anxiety or depression.

**Table 1.** *Outcome following amputation or limb salvage*

| Study | Sample (*n*) | Participant's age (years) | Report source | Controls | Site of tumour | Measures | Results |
|---|---|---|---|---|---|---|---|
| 7. Sugarbaker *et al.*, 1982 | *Study 1*. amputees: 9 limb salvage: 12<br>*Study 2*. amputees:8, limb salvage 15 | At diagnosis: <21 | Self | Norms | *Study 1*. upper and lower limb<br>*Study 2*. lower limb only | Sickness Impact Profile;[18] Activities of Daily Living Scale;[19] Function;[20] Psychosocial Adjustment to Illness Scale[21] | No substantial differences between the two groups on any measures |
| 8. Weddington *et al.*, 1985 | 14 amputees; 19 limb salvage | At surgery: 11–70 at time of study; 13–71 | Self | Norms | Upper and lower limb | Cognitive Capacity;[22] Hopkins Symptom Checklist;[21] Depression; [23] Mood;[24] Function;[25] Global Adjustment to Illness;[21] Schedule for Affective Disorders[26] | • No differences between amputees and limb salvage patients on indices of:<br>—cognitive capacity<br>—anxiety<br>—depressive symptoms<br>—adjustment to illness<br>• Scores on the lifetime prevalence for psychiatric disorders were 45% after surgery, compared with 25% before.<br>• Evidence of some major impact on depression and affective disorder in the whole group |
| 9. Nicholson *et al.*, 1992 | 82 | <20 at diagnosis | Self | 151 siblings | Not specified | Health, fertility, number of children, employment, income and daily living | Survivors were more likely than siblings to:<br>• experience difficulties climbing stairs<br>• have received employment disability payments<br>Siblings and survivors did not differ in:<br>• employment status<br>• annual income<br>• marriage rate and fertility |
| 10. Postma *et al.*, 1992 | 14 limb salvage; 19 amputees; 3 refusals | Limb salvage=24 (13–56 years)<br>Amputees=27 (21–53 years) | Self | Norms | Lower limb | Symptoms;[21] activity restrictions;[27] self-esteem;[28] interview (education, employment, marital status, costs of living, participation in social life) | • No differences between groups except that:<br>• LS reported more somatic symptoms, and<br>• amputees reported lower self-esteem |
| 11. Greenberg *et al.*, 1994 | 62 response rate=62 of 89 | On diagnosis: 6–67 at time of study: mean(age)=33 | Self | Norms | Upper and lower limb | Demographic information; ratings of: QoL before and after cancer, impact of cancer on education and income, sexual function, child-bearing and fertility; Psychosocial Adjustment to Illness;[21] Impact of Events;[29] Mood;[24] the MGH Function Questionnaire (Butterfield, unpublished observation); Limb Value Questionnaire (ref not given); Pain[30] | • Psychiatric symptoms; were similar to the general population<br>• Pain: 35% reported pain to be negligible; 44% mild; 5 reported pain to be their worst problem; 66% took no analgesics<br>• Fertility: 5 men and 8 women had successfully had children<br>• Function: 80% reported that they had resumed their previous activities; 20% resumed half their previous activities<br>• Limb salvage patients: successful limb salvage patients were more positive than unsuccessful patients<br>• Satisfaction remained high even among those who experienced amputation following a failed prosthesis |
| 12. Rougraff *et al.*, 1994 | 29 (8 limb-salvage; 5 hip disarticulation; 16 amputees) | <30 mean(age)=16 | Self | None | Lower limb | Demographic and cancer/treatment history information; Functional assessment;[31,32] Quality of life assessment; Psychosocial Adjustment to Illness;[21] Pain;[30] questionnaires on demographic aspects, fertility, & sexual function | • Functional assessment—limb-salvage patients had significantly higher function scores than amputees and hip-disarticulation patients<br>• Quality of life assessment—no differences between the three groups on the basis of quality of life data |

**Table 1**. *Continued*

| Study | Sample (*n*) | Participant's age (years) | Report source | Controls | Site of tumour | Measures | Results |
|---|---|---|---|---|---|---|---|
| 13. Christ *et al.*, 1995 | 32 limb salvage; 13 amputees response rate=45 of 65 | At diagnosis: 11–24 | Self | Norms | Lower limb | Interview; Brief Symptom Inventory;[21] Depression;[33] Self-Esteem;[28] coping (3 items of social support; 2 vocational measures); body-image[34] | • About 75% reported that they worked at their level of competence<br>• 19 felt that their illness adversely affected their career<br>• Amputees were less likely then limb salvage patients to have married since their diagnosis<br>• Psychological symptoms:<br>• No difference between patients' mean scores and population norms<br>• Amputees had non-significantly higher symptom and depression scores than limb salvage patients<br>• Limb salvage patients had significantly better coping scores than amputees |
| 14. Eiser *et al.*, 1997 | 41 limb salvage response rate=41 of 63. | Age at diagnosis=10.9 (5–14) at time of study=17.7 (8–25) | Self & mother | Norms | Lower limb | Survivors and mothers: interview (regarding impact on schooling, work, mobility, pain & body image, and emotional well-being);<br><br>Survivors: Functional Evaluation of Reconstructive Procedures;[32] Survivors 18 years+: Short Form 36 (SF36) health survey questionnaire[35] | • Mobility: 12% reported that they were able to run; 49% that they were able to swim; 46% able to ride a bike<br>• Schooling: many experienced lengthy school absence; 8 repeated a school year<br>• Emotional well-being: 3 patients were receiving psychiatric support<br>• Most believed that the prosthesis had given them opportunity for a better QoL than amputation<br>• SF36: scores were below population norms; scores on subscales measuring physical functioning, physical role performance, pain, general health, and social functioning were significantly different to norms<br>• Fertility: children had been born to 2 females and 1 male (to be expected from drug regimes and age of cohort) |
| 15. Novacovic *et al.*, 1997 | 89 | | | 97 siblings | | Education, employment, marriage, fertility, treatment related difficulties. Function[36] | No differences in educational achievement. Survivors less likely to be employed full-time, married or have children. Function poor compared with siblings |
| 16. Felder-Puig *et al.*, 1998 | 60 response rate=54%, 49 limb salvage and 11 amputation | Age at diagnosis= 15.27range=3 months-25 years; Age on testing=23.5 years, range=15–30 years | Self | | 'Extremities' | Selected parts of Subjective well-being;[37] Anxiety;[38] Self-concept;[39] Life goals;[40] Interview | No differences based on surgery, gender, time since treatment and recurrence.<br>Those diagnosed in adolescence had more problems than those diagnosed in childhood or early adulthood.<br>Non-respondents were more likely to have had amputation. |
| 17. Johansen *et al.*, 1998 | 58 amputees; 89 limb salvage | At time of study: LS=50, (14–87); amp=47 (14–88) age at surgery not given. | Self | | Upper and lower limb | Functional assessment[32] | Limb salvage patients had significantly higher functional score than amputees (85 vs 47) |

However, three of these studies reported that a number of individuals sought professional help to deal with emotional problems surrounding their surgery.[11,12,14]

**How does QoL differ between amputees, successful limb salvage and failed limb salvage?**

*Physical functioning*

Six studies reported comparisons between those treated by amputation and limb salvage. Physical function was reported to be better for the limb salvage group in two studies[12,17] and for the amputee group in one study.[13] Three studies reported no differences.[7,8,10] It should be noted that in two studies where a specific measure of function was used,[12,17] differences in favour of the limb salvage group were reported. A generic instrument may lack sensitivity in this context[7] and a non-standardized interview[13] may be less likely to yield reliable data.

Two studies assessed function only in patients with limb salvage.[11,14] Most (80%) were able to resume their previous activities. However, 16% could only walk 50–100 feet.[11] Difficulties running (88%), swimming (49%) and riding a bike (46%) were reported.[14]

*Pain*

Half of the patients interviewed by Greenberg *et al.*[11] reported pain, though most of these described negligible or mild pain. Eiser *et al.*[14] reported that 24% of a group of patients treated by limb salvage experienced no pain, 7% reported pain when it was cold, and 15% only experienced pain after strenuous exercise. Nineteen reported serious and continuous pain, with 10 of these reporting occasional or regular use of analgesics. Rougraff *et al.*[12] found that 25 of 29 patients reported no pain and there were no differences between the amputation and limb salvage groups.

*Psychological functioning*

Felder-Puig *et al.*[16] used standardized German measures to calculate indices of emotional well-being, social well-being, love life and subjective capabilities and reported no differences as a function of type of treatment. Similar results were found for measures of adjustment to illness, perceived impact of illness, and activities of daily living[7] and for mood, adjustment to illness or psychiatric symptoms.[8,10,12]

Based on interview data, Eiser *et al.*[14] reported that 25 of 41 patients treated by limb salvage were distressed about the physical appearance of their leg. A small number (mostly women) always chose clothes to hide their leg (long trousers or skirts).

*Social functioning*

Nicholson *et al.*[9] found no differences in employment, income or marriage rates compared with siblings, but all other reports have suggested compromised levels of functioning. Felder-Puig *et al.*[16] reported that patients were less likely to be married, more likely to remain at home with parents and less likely to have children compared with population statistics. Level of education was similar and incomes appeared comparable. However, 18% had given up work because of treatment, and 27% had changed their type of work. Many reported difficulties in obtaining work. Lower level of education, single marital status or lower salary were all seen to be a consequence of the illness.[12] Novakovic[15] did find that survivors (all Ewing's sarcoma) were less likely to be employed full-time, to be married or have children compared with siblings. However, there were no differences in insurance status or use of health care services. Amputees felt they were not employed at an appropriate level and they were also less likely to have married.[13] Amputees reported more work and social discrimination compared with the limb salvage group and were more socially isolated.[10] Fourteen of 40 limb salvage patients interviewed about their work felt that they were limited because of their illness, mostly those with lower education levels.[14]

Most studies do not provide data on sexual functioning. However, limb salvage patients reported greater reduction in sexual functioning compared with amputees.[7] Patients treated in the early days of limb salvage surgery often had stiff, painful or oedematous legs following radiotherapy, and direct comparisons with more recently treated patients should therefore not be attempted. Most patients had sexual experience and reported few problems with sexual functioning, but amputees were less likely than limb salvage patients to have married.[13] Clearly, these data are difficult to collect and self-reports may be subject to considerable bias.

Greenberg *et al.*[11] noted that patients generally were positive about limb salvage compared with amputation, and were prepared to spend months in hospital to try to preserve the limb. Patients were asked to rate on a five-point scale whether the efforts to save the limb had been worthwhile. Mean ratings were highest (indicating most positive ratings) for those with successful limb salvage (4.5) compared with those treated by amputation (3.6). Those who experienced amputation following failed limb salvage still rated the effort as very worthwhile (4.0). Only one of the patients actively chose amputation. Satisfaction remained high, even among those who experienced amputation following a failed prosthesis.[11] There was some opinion that the time involved in trying to save the limb allowed the patient the opportunity to adjust to the possibility of limb loss. In the context of failed limb salvage, patients also reported that it was important to make known their own preferences about amputation to surgeons, given the previous investment in preserving the limb.

Some recognized that surgeons were disappointed and therefore often reluctant to accept the need for amputation.

### Do young children adapt better to amputation than older children or adolescents?

The assertion that young children adapt quickly following amputation is common, but we found little evidence to support the view. 'Children quickly adapt to detachable limb prosthesis and after a short period acquire remarkable agility' (Pinkerton *et al.*[42], p.176). Circumstantial evidence is provided by Felder-Puig *et al.*[16], who reported that patients diagnosed as children (<13 years) had fewer problems (as measured by social well-being) than those diagnosed during adolescence (14–19 years). There was no comparable effect on their measure of physical well-being. No other paper addresses this question.

### Comment on methods

There are substantial difficulties inherent in conducting psychosocial work in this area. It is not possible to conduct randomized control trials and assign patients to different treatment options. Simple comparisons between populations differing in surgery are inevitably confounded by demographic and clinical differences between groups, which may contribute more to psychological difficulties than the surgical procedures of primary interest. In addition, conventional questionnaires do not adequately address the question. The development of appropriate and sensitive QoL measures for use with this population has to be a priority for future work. Without such measures, we lack tools to assess QoL outcomes between patients experiencing different treatments, or to evaluate any intervention programmes.

Given the above reservations, limitations in the current literature need to be recognized, some of which make interpretation of the findings problematic. First, participation rates are often less than ideal, and this raises questions about the representativeness of findings. This partly reflects relatively poor survival rates, particularly in Ewing's tumour, and partly also difficulties in tracing patients who have not been involved in active follow-up many years after treatment. The possibility that those who are traced and are willing to be involved have a different quality of life from those who cannot be located or do not respond to requests to participate, has to be considered.

Probably for a number of reasons, information about non-participants is rarely reported. Where it is available, it is invariably limited to brief demographic or clinical variables. Based on such analyses, most studies report no differences between those who do and do not participate.[8,13,14] These comparisons are based on very broad and often initial indicators of disease and may not reflect ongoing differences between the groups. More detailed follow-up was provided by Felder-Puig *et al.*[16] who followed up non-participants by phone. Non-participants were more likely to be amputees, more likely to be depressed and had higher rates of substance abuse and legal difficulties compared with participants. In a separate study, refusers were more likely to be male, and to have higher rates of substance abuse and legal difficulties.[11]

Second, a relatively large number of outcome measures have been employed. This reflects the lack of suitable instruments available and failure to specify the ways in which quality of life may be most likely to be affected. We need to move away from functionally orientated QoL measures and adopt more process orientated models that recognize patients' capacity to re-appraise their lives and adjust to their current health status.[43]

Third, very few studies have included patients who were treated as children. Patients were diagnosed before 20, 21 and 24 years, respectively;[7,9,13] the sample described by Eiser *et al.*[14] included those diagnosed between 5 and 14 years. In other cases, some young children and adolescents were included, but for analyses all patients regardless of age are treated as a homogenous group. This approach fails to address the specific issues that affect children and young people following amputation or limb salvage surgery, relating especially to issues of body image, mobility and social functioning. It is one thing to lose a leg when established in work and social relationships; it is quite another if, as an adolescent, it is necessary to negotiate entrance to the adult world of work and social relationships. Outcome measures need to be sensitive to developmental issues. There is no empirical evidence regarding how well children adapt to surgery compared with adults. This kind of work is needed in order to develop appropriate physiotherapy and promote successful adaptation among younger patients and to consider service needs for child patients.

Fourth, in many studies sample sizes may be so small that it would not be possible to identify differences between groups. Calculations of effect sizes should be made so that it is clear that the sample is large enough that any underlying differences can be identified. In practice, it may be naive to make simple comparisons between groups who have been treated by limb salvage or amputation. Limb salvage is invariably the treatment of choice. This means that patients who experience an amputation are likely to have first experienced many months of limb salvage. Acceptance of the amputation may therefore be influenced by their experiences of limb salvage. While some may interpret the need for amputation as a failure, others will see the earlier experience as essential in aiding adjustment to the loss of the limb. We would like to reiterate our point that these are not questions that can be answered by adopting conventional experimental designs, but require more innovative research methods.

Fifth, there are difficulties in interpreting studies involving comparisons of employment, education level or marital status, either against population statistics or between treatment groups. To any independent observer, it is clear that treatment for a bone tumour, whether amputation or limb salvage, will adversely affect an individual's job prospects. The fact that more patients do not report difficulties is a testament to individual resilience and adaptability.

### Conclusions

Given the importance of failed limb salvage, it is disappointing that so little research has attempted to determine the psychological impact for the patient and the family of failed limb salvage surgery. Above all, this situation requires a good relationship between patient and surgeon, in that both must recognize the need to maximize function and improve QoL. Either the patient or the surgeon may assume that the other has more invested in preserving the limb. Considerable sensitivity is called for in opening negotiations about amputation, and patients may need considerable time to adjust to the need for further surgery. Contrary to the view that failed limb salvage will be emotionally problematic for patients, it may well be that better outcomes are possible where patients feel they made their own decision about surgery.

One of the merits of the systematic review method is the identification of gaps in the available literature. We include in this the lack of attention to the specific needs of children and their families. Without exception, research has included patients several years after their initial treatment. This means that we know little about the initial impact of treatment, how patients adapt to their surgery or how they set about rebuilding their lives. Although there is an assumption that surgery affects body image, this has rarely been systematically assessed.

The issue of gender differences in adjustment has not been consistently addressed. Any chronic illness which limits physical functioning may be more detrimental for the QoL of males compared with females, since it might compromise opportunities for males to participate in team sports and aggressive games. In contrast, amputation may have a worse impact on QoL for females, assuming the greater importance of body image.[13,14] Females appeared to adjust as well as males, though they also reported more anxiety about how others might react to them.[13] Differences are not likely to be limited to participation in sports, but comprehensive assessment must include a range of activities, including social as well as physical functioning. In contemporary society and our world of 'equal opportunities', we perhaps should not expect that the consequences of surgery will be related to gender. However, we need to acknowledge a substantial body of literature that attests to differences in emotional response and coping preferences following trauma between men and women.[44]

The results of follow-up studies describing the QoL of survivors of bone tumours is potentially useful in order to provide information to new patients about likely outcomes and the expected course of events. For surgeons, such information may aid decision making and guide the way in which information is presented to new patients. The papers reviewed provide some information about physical and psychological outcomes, although interpretation needs to be made with care, given the methodological limitations inherent in most work.

Although there have been a number of studies describing physical and psychological outcomes following treatment for bone tumour, little is directly relevant to clinical issues. Questions such as how well young children adapt following amputation, or the effects of failed limb salvage have been rarely addressed. It is important that future research addresses these issues directly rather than focus generally on psychosocial adjustment.

### Acknowledgement

This work was funded by the Cancer Research Campaign (CP1019/0101 and CP1019/0401).

### References

1 Hawkins MM, Stevens MCG. The long term survivors. *BMJ* 1996;52:898–923.
2 Bramwell VH, Burgers M, Sneath RS, *et al.* A comparison of two short intensive adjuvant chemotherapy regimens in operable osteosarcoma of limbs in children and young adults: the first study of the European Osteosarcoma Intergroup. *J Clin Oncol* 1992;10:1579–91.
3 Feinstein AR. Clinimetric perspectives. *J Chronic Disorders* 1987;40:635–40.
4 Reaman GH, Haase GM. Quality of life research in childhood cancer: the time is now. *Cancer* 1996;78:1330–2.
5 Rosenbaum P, Cadman D, Kirpalani H. Pediatrics: Assessing quality of life. In: Spilker B, ed. *Quality of Life Assessments in Clinical Trials.* New York: Raven Press, 1990.
6 Leplege A, Hunt S. The problem of quality of life in medicine. *JAMA* 1997;278:47–50.
7 Sugarbaker PH, Barofsky I, Rosenberg SA, Gianola FJ. Quality of life assessment of patients in extremity sarcoma clinical trials. *Surgery* 1982;91:17–23.
8 Weddington WW, Segraves KB, Simon MA. Psychological outcome of extremity sarcoma survivors undergoing amputation or limb salvage. *J Clin Oncol* 1985;3:1393–9.
9 Nicholson HS, Mulvihill JJ, Byrne J. Late effects of therapy in adult survivors of osteosarcoma and Ewing's sarcoma. *Med Ped Oncol* 1992;20:6–12.
10 Postma A, Kingma A, De Ruiter JH, Schraffordt KH, Veth RP, Goeken LN, Kamps WA. Quality of life in bone tumor patients comparing limb salvage and amputation of the lower extremity. *J Surg Oncol* 1992;51:47–51.

11 Greenberg DR, Goorin A, Gebhardt MC, Gupta L, Stier N, Harmon D, Mankin H. Quality of life in osteosarcoma survivors. *Oncol* 1994;8:19–25.

12 Rougraff BT, Simon MA, Kneisl JS, Greenberg DB, Mankin HJ. Limb salvage compared with amputation for osteosarcoma of the distal end of the femur. *Am J Bone Joint Surg* 1994;76:649–56.

13 Christ GH, Lane JM, Marcove R. Psychosocial adaptation of long-term survivors of bone sarcoma. *J Psychosoc Oncol* 1995;13:1–21.

14 Eiser C, Cool P, Grimer RJ, Carter SR, Cotter IM, Ellis AJ, Kopel S. Quality of life following treatment for a malignant primary bone tumour around the knee. *Sarcoma* 1997;1:39–45.

15 Novakovic B, Fears TR, Horowitz ME, Tucker MA, Wexler LH. Late effects of therapy in survivors of Ewing's sarcoma family tumors. *J Ped Hem/Oncol* 1997;19:220–5.

16 Felder-Puig R, Formann AK, Mildner A, Bretschneider W, Bucher B, Zoubek A, Puig S, Topf R. Quality of life and psychosocial adjustment of young patients after treatment of bone cancer. *Cancer* 1998;83:69–75.

17 Johansen R, Nielsen OS, Keller J. Functional outcome in sarcomas treated with limb salvage surgery or amputation. *Sarcoma* 1998;2:19–23.

18 Bergner M, Bobbit RA, Pollard WE, Gilson BS. The sickness impact profile: validation of a health status measure. *Med Care* 1976;14:57–67.

19 Katz S, Akpom CA. A measure of primary sociobiological functions. *Int J Health Serv* 1976;6:493–507.

20 Mahoney FI, Bartel DW. Functional evaluation: the Bartel index. *Md State Med J* 1965;14:64–5.

21 Derogatis LR. The psychosocial adjustment to illness scale. *J Psychosomat Res* 1986;30:77–91.

22 Jacobs JW, Bernhard MR, Delgado A. Screening for organic mental syndromes in the medically ill. *Ann Intern Med* 1977;86:40–6.

23 Beck AT. *Depression: Clinical, Experimental, and Theometric Aspects*. New York: Harper & Row, 1997.

24 Mcnair D, Lorr M, Droppelman L. *Psychiatric Outpatient Mood Scale*. Boston: Psychopharmacology Laboratory, Boston Medical Centre, 1971.

25 Karnofsky DA, Burchenal JH. Clinical evaluation of chemotherapeutic agents in cancer. In: MacLeod CM, ed. *Evaluation of Chemotherapeutic Agents*. New York: Columbia University, 1949:33–8.

26 Endicot J, Spitzer RL. A diagnostic interview: The schedule for affective disorders and schizophrenia. *Arch Gen Psychiatry* 1978;35:837–44.

27 Kempen GIJM, Suurmeijer TPBM. The development of a hierarchical polychotomous ADL-IADL scale for non-institutionalised elders. *Gerentologist* 1990;30:497–502.

28 Rosenberg M. *Society and the Adolescent Self-image*. Princeton, New Jersey: Princeton University Press, 1965.

29 Horowitz M, Wilner N, Alvarez W. Impact of event scale: a measure of subjective stress. *Psychosomatic Medicine* 1979;41:209–18.

30 Melzack R. The McGill pain questionnaire: major properties and scoring methods. *Pain* 1975;1:277–99.

31 Enneking WF, Dunham W, Gebhaedt MC. A system for the functional evaluation of reconstructive procedures after surgical treatment of tumors of the musculoskeletal system. *Clin Orthop Related Res* 1993;99:480–3.

32 Insall JN, Dorr LD, Scott RD, Scott WN. Rationale of the Knee Society clinical rating system. *Clin Orthop* 1989;248:13–14.

33 Radloff LS. *CES-D a measure of depression*. Bethesda, MD: National Institute of Mental Health, Centre for Epidemiological Studies in Depression, 1977.

34 Derogatis L, Melisaratos N. The DSFI: multidimensional measure of sexual functioning. *J Sex Marital Therapy* 1979;5:244–58.

35 Jenkinson C, Coulter A, Wright L. Short form SF 36 health survey questionnaire: normative data for adults of working age. *BMJ* 1993;306:1437–40.

36 Karnofsky DA, Abelman WH, Craver LF *et al.* The use of nitrogen mustards in palliative treatment of carcinoma. *Cancer* 1948;1:634–56.

37 Grob A, Luethi R, Kaiser FK *et al. Berner Fragebogen zum Wohlbefinden Jugendlicher: Entwicklung und Ueberpruefung*. Forschungsbericht aus dem psychologischen Institut der Universitaet Bern. Bern: Universitaet Bern, 1989.

38 Laux L, Glanzmann P, Schaffner P, Spielberger CD. *Das State-Trait Angstinventar*. Weinheim: Beltz, 1981.

39 Hassan Deusinger IM. *Die Frankfurter Selbstkonzeptskalen*. Goettingen: Hogrefe, 1986.

40 Kraak B, Nord-Ruediger D. Fragebogen zu Lebenszielen und zur Lebenszufriedenheit. *Med Ped Onc* 1996;27:232.

41 Grimer RJ, Carter SR, Pynsett PB. The cost-effectiveness of limb salvage for bone tumours. *Br J Bone Joint Surg* 1997;79B:558–61.

42 Pinkerton CR, Cushing P, Sepion B. *Childhood Cancer Management*. London: Chapman & Hall Medical, 1994.

43 Bury M. The sociology of chronic illness. *Social Health Illness* 1991;13:451–68.

44 Hobfoll SE. Gender differences in stress reactions: Women filling the gap. *Psychol Health* 1991;5:95–110.
